# Comparison of international guideline programs to evaluate and update the Dutch program for clinical guideline development in physical therapy

**DOI:** 10.1186/1472-6963-7-191

**Published:** 2007-11-23

**Authors:** Philip J Van der Wees, Erik JM Hendriks, Jan WH Custers, Jako S Burgers, Joost Dekker, Rob A de Bie

**Affiliations:** 1 Department of Epidemiology, Center for Evidence based Physiotherapy and Caphri Research Institute, Maastricht University, Maastricht, The Netherlands; 2 Royal Dutch Society for Physical Therapy (KNGF), Amersfoort, The Netherlands; 3 Dutch Institute for Allied Health Care (NPi), Amersfoort, The Netherlands; 4 Dutch Institute for Health Care Improvement (CBO), Utrecht, The Netherlands; 5 Department of Rehabilitation Medicine, EMGO Institute, VU University Medical Centre, Amsterdam, The Netherlands

## Abstract

**Background:**

Clinical guidelines are considered important instruments to improve quality in health care. Since 1998 the Royal Dutch Society for Physical Therapy (KNGF) produced evidence-based clinical guidelines, based on a standardized program. New developments in the field of guideline research raised the need to evaluate and update the KNGF guideline program.

Purpose of this study is to compare different guideline development programs and review the KNGF guideline program for physical therapy in the Netherlands, in order to update the program.

**Method:**

Six international guideline development programs were selected, and the 23 criteria of the AGREE Instrument were used to evaluate the guideline programs. Information about the programs was retrieved from published handbooks of the organizations. Also, the Dutch program for guideline development in physical therapy was evaluated using the AGREE criteria. Further comparison the six guideline programs was carried out using the following elements of the guideline development processes: Structure and organization; Preparation and initiation; Development; Validation; Dissemination and implementation; Evaluation and update.

**Results:**

Compliance with the AGREE criteria of the guideline programs was high. Four programs addressed 22 AGREE criteria, and two programs addressed 20 AGREE criteria. The previous Dutch program for guideline development in physical therapy lacked in compliance with the AGREE criteria, meeting only 13 criteria.

Further comparison showed that all guideline programs perform systematic literature searches to identify the available evidence. Recommendations are formulated and graded, based on evidence and other relevant factors. It is not clear how decisions in the development process are made. In particular, the process of translating evidence into practice recommendations can be improved.

**Conclusion:**

As a result of international developments and consensus, the described processes for developing clinical practice guidelines have much in common. The AGREE criteria are common basis for the development of guidelines, although it is not clear how final decisions are made. Detailed comparison of the different guideline programs was used for updating the Dutch program. As a result the updated KNGF program complied with 22 AGREE criteria. International discussion is continuing and will be used for further improvement of the program.

## Background

Development and implementation of evidence-based clinical guidelines are the main focus of health care policy in many countries. Clinical guidelines are 'systematically developed statements designed to help practitioners and patients to make decisions about appropriate health care for specific circumstances' [1]. Also in physical therapy clinical guidelines are considered important instruments to improve quality of care. Since 1998 the Royal Dutch Society for Physical Therapy (KNGF) has produced evidence-based clinical guidelines [2]. KNGF-guidelines were developed using a standardized procedure [3,4]. However, new developments in the field of guideline research raised the need to evaluate and update the current program.

An important reason for updating the development process was the publication of the AGREE Instrument which provides a framework, including 23 criteria, to assess the quality of clinical practice guidelines [5]. The instrument is based on international consensus about methods for developing evidence-based clinical guidelines [6,7]. It also helps guideline developers to structure and improve the process of guideline development. The Dutch network of guideline organizations adopted the AGREE Instrument, and reached consensus in methods for assessing and synthesizing the evidence [8,9]. An international survey of 18 clinical guideline programs also showed a growing international consensus on the structure and working methods of those programs [6]. In 2002 the Guidelines International Network (G-I-N) was founded to promote systematic development of clinical guidelines [10]. The World Confederation for Physical Therapy (WCPT) also published frameworks for guideline development based on international developments [11-13]. Further refinement of the guideline development process is currently subject of international debate. The Advisory Committee on Health Research of the World Health Organization (WHO) has conducted a series of reviews to advise on ways to improve the use of research evidence in guidelines [14].

One of the key issues in the debate is to formulate criteria for inclusion of considerations beyond the evidence from literature reviews [15-17].

The aim of this study is to compare a selection of different guideline development programs in detail and to use these findings to review the previous KNGF guideline program in the Netherlands. The results are used to update the KNGF guideline program.

## Methods

### Study design

Burgers [6] defined a guideline program as a structured and coordinated program, designed with the specific aim of producing several clinical practice guidelines. Based on an international survey of 18 clinical guideline programs by Burgers [6] we selected guideline programs from five organizations: the Dutch Institute for health care improvement (CBO), Netherlands [18]; the National Health and Medical Research Council (NHMRC), Australia [19]; the New Zealand Guidelines Group (NZGG), New Zealand [20]; the Scottish Intercollegiate Guidelines Network (SIGN), Scotland [21]; and the U.S. Preventive Services Task Force (USPSTF), USA [22,23]. Criterion for selection was a publicly available handbook for the development of guidelines, written in English or Dutch. Four of these programs [19-23] were also included in the review of Schünemann [24], in which guidelines for the development of guidelines were reviewed by the WHO Advisory Committee on Health Research. In addition we searched databases of the Guidelines International Network [25], the National Guideline Clearinghouse [26] and the World Confederation for Physical Therapy [27], to identify guideline programs specifically aimed at physical therapy. This resulted in the addition of a sixth guideline program, published by the Chartered Society of Physiotherapy (CSP) [28]. No other physical therapy guideline programs were identified. Basic characteristics of the selected guideline programs are shown in Table 1.

**Table 1:** Basic characteristics of guideline programs

**Organization**	**Country**	**Year of first guideline**	**Total guidelines**
CBO [18] Dutch Institute for health care improvement	Netherlands	1980	> 50
CSP [28] Chartered Society of Physiotherapy	England	1998	5–10
NHMRC [19] National Health and Medical Research Council	Australia	1995	10–20
NZGG [20] New Zealand Guidelines Group	New Zealand	1998	> 50
SIGN [21] Scottish Intercollegiate Guidelines Network	Scotland	1995	> 50
USPSTF [22,23] U.S. Preventive Services Task Force	USA	1989	> 50

Source: Burgers [6], added and updated with own data via handbooks and websites

### Data collection and analysis

The 23 criteria of the AGREE instrument [5] were used to evaluate the guideline programs, reviewing published handbooks of the six organizations. If necessary, additional information was retrieved from the websites of the organizations. The handbooks were screened by two reviewers to analyze whether the AGREE criteria were addressed in the guideline development process, resulting in positive (+) or negative (-) judgment. Disagreement between reviewers was followed by discussion, in order to reach consensus. The previous Dutch program for guideline development in Physical Therapy was also evaluated using the AGREE criteria, to identify weaknesses that could lead to improvements of the program.

We further compared the different guideline programs in detail based on the guideline structure by Shekelle [7]. It includes the following elements: (1) Structure and organization; (2) Preparation and initiation; (3) Development; (4) Validation; (5) Dissemination and implementation; (6) Evaluation and update. Information from the handbooks was systematically extracted by one reviewer, and checked by a second reviewer. Disagreement between reviewers was followed by discussion, in order to reach consensus.

## Results

### Compliance with the AGREE criteria

Compliance with the AGREE criteria in the descriptions of the guideline development process was high. Four organizations (CBO, NHMRC, NZGG, SIGN) complied with 22 out of 23 criteria. The other two organizations (CSP, USPSTF) complied with 20 criteria.

The criterion least met was 'The guideline has been piloted among target users'. In the handbooks of four organizations (CBO, NHMRC, SIGN, USPSTF) piloting of the guideline was not specifically described or recommended. Table 2 shows an overview of compliance to the AGREE criteria in the handbooks of the six organizations.

**Table 2:** Compliance to AGREE criteria of six guideline development programs

**AGREE criteria**	**CBO**	**CSP**	**NHMRC**	**NZGG**	**SIGN**	**USPSTF**
1. The overall objective(s) of the guideline is (are) specifically described.	+	+	+	+	+	+
2. The clinical question(s) covered by the guideline is (are) specifically described.	+	+	+	+	+	+
3. The patients to whom the guideline is meant to apply are specifically described.	+	+	+	+	+	+
4. The guideline development group includes individuals from all the relevant professional groups.	+	+	+	+	+	-
5. The patients' views and preferences have been sought.	+	+	+	+	+	-
6. The target users of the guideline are clearly defined.	+	+	+	+	+	+
7. The guideline has been piloted among target users.	-	+	-	+	-	-
8. Systematic methods were used to search for evidence.	+	+	+	+	+	+
9. The criteria for selecting the evidence are clearly described.	+	+	+	+	+	+
10. The methods used for formulating the recommendations are clearly described.	+	+	+	+	+	+
11. The health benefits, side effects and risks have been considered in formulating the recommendations.	+	+	+	+	+	+
12. There is an explicit link between the recommendations and the supporting evidence.	+	+	+	+	+	+
13. The guideline has been externally reviewed by experts prior to its publication.	+	+	+	+	+	+
14. A procedure for updating the guideline is provided.	+	+	+	+	+	+
15. The recommendations are specific and unambiguous.	+	+	+	+	+	+
16. The different options for management of the condition are clearly presented.	+	+	+	+	+	+
17. Key recommendations are easily identifiable.	+	-	+	+	+	+
18. The guideline is supported with tools for application.	+	-	+	+	+	+
19. The potential organizational barriers in applying the recommendations have been discussed.	+	+	+	+	+	+
20. The potential cost implications of applying the recommendations have been considered.	+	+	+	+	+	+
21. The guideline presents key review criteria for monitoring and/or audit purposes.	+	-	+	-	+	+
22. The guideline is editorially independent from the funding body.	+	+	+	+	+	+
23. Conflicts of interest of guideline development members have been recorded.	+	+	+	+	+	+

### Evaluation of the Dutch guideline program in physical therapy

To evaluate the previous Dutch guideline program in physical therapy we also used the AGREE criteria. Identification of weaknesses could then subsequently be used to update the program. In Table 3 compliance with the AGREE criteria of the previous Dutch program for clinical guideline development in Physical Therapy is shown. In the previous program only 13 AGREE criteria were met. Compliance was mainly lacking in specific and systematic formulation of recommendations, based on evidence and other considerations. Also, the previous Dutch program did not provide a procedure for updating guidelines.

**Table 3:** Compliance to AGREE criteria of previous and updated KNGF guideline program

**AGREE criteria**	**KNGF (previous)**	**KNGF (update)**
1. The overall objective(s) of the guideline is (are) specifically described.	+	+
2. The clinical question(s) covered by the guideline is (are) specifically described.	+	+
3. The patients to whom the guideline is meant to apply are specifically described.	+	+
4. The guideline development group includes individuals from all the relevant professional groups.	+	+
5. The patients' views and preferences have been sought.	+	+
6. The target users of the guideline are clearly defined.	+	+
7. The guideline has been piloted among target users.	-	-
8. Systematic methods were used to search for evidence.	+	+
9. The criteria for selecting the evidence are clearly described.	+	+
10. The methods used for formulating the recommendations are clearly described.	-	+
11. The health benefits, side effects and risks have been considered in formulating the recommendations.	-	+
12. There is an explicit link between the recommendations and the supporting evidence.	-	+
13. The guideline has been externally reviewed by experts prior to its publication.	+	+
14. A procedure for updating the guideline is provided.	-	+
15. The recommendations are specific and unambiguous.	-	+
16. The different options for management of the condition are clearly presented.	+	+
17. Key recommendations are easily identifiable.	-	+
18. The guideline is supported with tools for application.	+	+
19. The potential organizational barriers in applying the recommendations have been discussed.	+	+
20. The potential cost implications of applying the recommendations have been considered.	-	+
21. The guideline presents key review criteria for monitoring and/or audit purposes.	-	+
22. The guideline is editorially independent from the funding body.	+	+
23. Conflicts of interest of guideline development members have been recorded.	-	+

### Comparison of Guideline Development Programs

#### Structure and Organization

Table 4 shows a comparison of the guideline programs based on the six elements of the development process. Five programs are coordinated by a central organization responsible for developing the guidelines. CSP used to endorse guidelines by other groups, as described in the handbook, but started developing guidelines in their own organization recently. Three programs (CSP, NHMRC, NZGG) endorse guidelines developed by other organizations within their country.

**Table 4:** Comparison of guideline programs

	CBO	CSP	NHMRC	NZGG	SIGN	USPSTF
**Structure and Organization**						
Central coordination of development and program	+	-	+	+	+	+
Endorsement of guidelines developed by others	-	+	+	+	-	-
**Preparation/initiation**						
Topics collected through open procedure	-	-	-	-	+	+
Criteria described for selection of topics	+	+	+	+	+	+
**Development**						
Quantitative studies considered for analysis	+	+	+	+	+	+
Qualitative studies considered for analysis	+	+	-	+	+	-
Evidence tables produced to critically appraise studies	+	+	+	+	+	+
Strength of evidence graded in levels of hierarchy	+	+	+	+	+	+
Recommendations graded in levels linked to evidence	+	+	+	+	+	+
**Validation/external review**						
Review by referees/stakeholders	+	+	+	+	+	+
Open meeting/conference	+	-	+	-	+	-
Consultation of practitioners (peers)	+	+	+	+	+	-
Draft guideline available on website for comments	+	-	-	-	+	-
**Dissemination/implementation**						
Publication on website	+	+	+	+	+	+
Full guideline published	+	+	+	+	+	+
Summary published	+	-	-	+	+	+
Patient version published	-	-	-	+	-	-
**Evaluation and revision**						
Timescale for updating guidelines is stated	+	+	+	-	+	+
Procedure for updating provided	+	+	+	+	+	+
Steps in updating procedure described	+	-	+	-	+	+
Continuous updating procedure available	+	-	-	-	-	-

#### Preparation/Initiation

Two organizations collect topics through an open procedure (SIGN, USPSTF). Any group or individual may propose a guideline topic to SIGN. USPSTF solicits new topics for consideration from the field through a periodic notice and solicitation of professional liaison organizations. All programs describe criteria for selecting topics. These include: clinical relevance, the number of patients affected, undesired variation in healthcare practice, no existing guidelines available, available evidence to support the guidelines, acceptability of a guideline to potential users. NZGG uses a suitability screen to assess the potential success of a guideline in a particular clinical area. Their key criterion is the ability to demonstrate significant positive changes in outcomes, based on valid scientific studies.

USPSTF prioritizes topics using two specific criteria: the public health importance of the condition to be prevented, and the potential for the USPSTF to affect clinical practice.

#### Development

The actual development of a guideline can be divided into four steps: refining subject area and defining questions; identifying the evidence available; assessing and synthesizing the evidence; and translating evidence into recommendations.

Three programs (CBO, CSP, USPSTF) use the scope of the guideline as a framework to refine the subject area and to formulate or refine research questions. NZGG develops a series of key questions using PECOT (Patient-Exposure-Comparison-Outcome-Time).

Identification of the evidence is done by systematic literature search. All programs stress the importance of a well-defined search strategy as part of a systematic review to be performed. Medline, Cinahl, Embase, Cochrane, PEDro are examples of databases used for searching relevant literature.

The details in assessing the literature vary among the programs, although the approach is similar. All programs perform systematic literature reviews, in which the outcomes of relevant studies are described in evidence tables and related to the methodological quality of the study. The strength of the evidence is classified in levels of evidence, but the classification varies. Four programs explicitly mention the inclusion of qualitative studies in the literature review (CBO, CSP, NZGG, SIGN).

The evidence from the studies is used to formulate recommendations, and recommendations are linked to the evidence by grading the recommendations in different levels. Beyond the evidence, other factors are considered in all programs, although USPSTF refrains from making recommendations if they cannot be supported by evidence. SIGN uses the concept of 'considered judgment' to cover these factors. A specific form is used to define specific recommendations considering changes to current practice, predicted impact on changes to current practice and economic issues and implications. NHMRC requires balancing of benefits and harms, to assist in formulating recommendations. Table 5 shows an overview of criteria and process of considered judgments by the different programs.

**Table 5:** Considered judgment to formulate recommendations

**Organization**	**Criteria for considered judgment**	**Process**
CBO	Clinical relevance Safety Patient perspective Professional perspective Available resources Cost-effectiveness Organization of care Legal consequences Ethical considerations Commercial interest	Considered judgment is described after description of the evidence. Formulation of the recommendation is based on the evidence and the considered judgment. A checklist is available for the development group with detailed criteria within ten domains as listed in this table.
CSP	Strength of evidence Clinical relevance and applicability of evidence Acceptability to patients Benefits and risks Costs	Development group should discuss considerations. When possible quantitative analysis should be made to estimate relative risks and benefits. Guideline document should describe some of the discussion and clearly describe the link between evidence review and recommendations.
NHMRC	Applicability of the evidence Probable outcome of intervention Balance of benefits against risks Alternative interventions Economic appraisal	A balance sheet is described to balance benefits and harms.
NZGG	Volume of evidence Consistency of the evidence Applicability of the evidence Clinical impact of the intervention	A considered judgment form is used to link clinical questions, evidence and recommendation. The development group needs to make a decision at the beginning of the process about how to resolve differences.
SIGN	Quantity, quality and consistency of evidence Generalizability of study findings Directness of application to target population Clinical impact Implementability	Development group summarizes view of considered judgment using a form to record their main points. The level of evidence is assigned to the judgment and a graded recommendation is formulated.
USPSTF	Quality of studies, linkage to key question using 3 criteria (internal validity, external validity, consistency), linkage to entire preventive service Magnitude and weighing of benefits and harms Extrapolation and generalization Other issues as cost effectiveness, resource prioritization, logistical factors, ethical and legal concerns, patient and societal expectations should be considered, but recommendations reflect primarily the state of the evidence.	Guideline topic team assesses criteria using systematic methods and rating systems. Recommendations reflect primarily the state of evidence. Making recommendations is done with the understanding that clinicians and policymakers must still consider additional factors in making their own decisions. Setting priorities in clinical practice (e.g. based on resource requirements) are beyond the scope of the review.

#### Validation

All programs organize an external review of draft guidelines by experts and stakeholders. Only two organizations (CBO, SIGN) publish draft guidelines on their website for comments.

#### Dissemination and implementation

All programs publish the full guidelines on their websites. NZGG is the only organization that publishes separate patient versions of the guideline.

#### Evaluation and revision

All programs describe the need for evaluation and revision of guidelines, but the steps of the updating process are not described in detail. Five programs provide a date for review in the guideline document. Length of update time varies depending on the topic and likelihood of new evidence. SIGN recommends assessment after two years, while CBO uses a five-year period.

CBO describes the concept of 'living guidelines', which follows a yearly cycle of reviewing the scope and clinical questions, performing an updated literature search identifying new evidence, and considering new factors and barriers in practice.

## Discussion

### Limitations of the study

The main purpose of this study was to compare different guideline programs and review the previous KNGF program for guideline development in physical therapy, based on an evidence-based approach. We collected information from handbooks and websites from six organizations that published guideline programs. We did not perform a random search for guideline programs, but based the selection on a previous survey by Burgers [6].

Comparing methodology for guideline development from handbooks, using the AGREE criteria, does not imply that the clinical guidelines themselves actually meet the AGREE criteria as well. Comparison of the published clinical guidelines was beyond the scope of this study.

### Compliance with AGREE criteria

All programs showed high compliance with the AGREE criteria, by means of addressing the criteria in the handbooks that describe the development process. Four of the six programs (CBO, CSP, NHMRC, NZGG) also explicitly mention the AGREE criteria as basis for their development process. The somewhat lower CSP score can be explained by their choice not to include steps for implementation in the handbook. The other lower score by USPSTF can be explained by their explicit choice to formulate recommendations strictly based on evidence, and not specifically involving the views of professionals and patients in the recommendations. Considerations beyond scientific evidence are not included in the scope of the reviews by USPSTF.

The previous Dutch program for guideline development in physical therapy complied only with 13 of the 23 AGREE criteria. These findings supported the need for updating the Dutch program. The lack of compliance with the AGREE criteria can partly be explained by the publication date in 1998, which was prior to publication of the AGREE instrument. Some changes were made in the development process, but were not yet published in an update of the handbook.

### Comparison of guideline programs

Our study confirms the findings of Burgers *et al.*[6] that guideline programs share basic principles, but differ in details. Although the collected information provided a lot of details about the development process, it is often not clear how decisions in the development process are made. For instance, a list of criteria is used for topic selection in most programs, but it is unclear how final decisions about the topic are made. Oxman et al. arrived at the same conclusion [29]. The difficulty of decision-making is also reflected in the formulation of recommendations. All programs describe the process for formulating recommendations in detail, supported by balance sheets or considered judgment forms. For example, Verkerk et al. presented a list of 37 items grouped into ten domains for considered judgment, which is used in the CBO program [30]. The final decision in formulating recommendations depends on discussions and consensus (formal and/or informal) within the guideline development group, weighing those different aspects in particular when evidence is lacking or contradictory [31,32].

All programs describe a procedure for weighing the evidence and grading recommendations using a hierarchy of levels of evidence. However, a large debate about the use of hierarchy levels is ongoing in the international guideline community [33,34]. Different grading systems exist and become more complicated, which hampers comparability. The international GRADE group has recently introduced and piloted a new system for grading the quality of evidence and the strength of recommendations [33,35]. The World Health Organization (WHO) adopted this system for their guidelines [36].

### Update of the Dutch guideline program for physical therapy

The update of the Dutch guideline program for physical therapy was based on evaluation of the current procedure and new insights. The framework of the updated Dutch program is shown in Table 6. The introduction of the AGREE Instrument and consensus by the Dutch Network resulted in several changes in the procedure of guideline development a few years ago. This concerned the inclusion of hierarchy of the evidence and grading of recommendations according to the Dutch network [8,9]. However, other aspects of the program needed further evaluation in order to make explicit choices. We focused on several aspects of the protocol: organizational structure; procedure for topic selection and defining the scope of guidelines; patient involvement; formulation of recommendations and a procedure for updating guidelines. From analyzing the several guideline programs we concluded that central coordination is needed to ensure a structured and systematic approach.

**Table 6:** Updated Dutch program for guideline development in physical therapy

**Section**	**Description**
1. Structure and organization	Central professional organization in collaboration with other institutes. Mono-disciplinary development group (5–10 members). Small group (2–3) of employed staff within development group responsible for review of literature and actual writing of the guideline. Patients involved in external review group and focus groups.
2. Preparation/initiation	Special interest groups can propose topics using application form. Procedure is described for prioritizing topics. Guideline committee selects. KNGF board makes final decision. Literature orientation on the subject. Barriers and needs of physiotherapists and patients are described in application form.
3. Development	Literature search using systematic strategy. Systematic review or meta-analysis if no (recent) review is available. Quality of studies assessed using different tools for diagnosis, intervention and systematic reviews. Hierarchy of the evidence described in four levels according to Dutch consensus. Grading of recommendations in four levels. Standardized formulation of recommendations according to grading. Outline of guideline divided in physical therapy diagnosis and treatment based on clinical reasoning process. Use of International Classification of Functioning (ICF) as nomenclature.
4. Validation	Draft guideline sent to group of peers to test practicality, clarity and acceptability. Draft guideline discussed in external review group (relevant health care professionals, patients, stakeholders). Separate check by patient advisory board. Final draft checked by Guideline Committee. Endorsement by KNGF. Test piloting after endorsement.
5. Dissemination and Implementation	Four products: practice guideline, review of the evidence, summary (in flow chart), patient version. Publication as supplement of Dutch Journal for Physical Therapy. Sent to all members of KNGF. Translated into English. Publication on website. Implementation plan with every guideline.
6. Evaluation and update	No later than 5 years after publication decision about update, based on new evidence, results from pilot, professional developments and developments in guideline methodology. Additional (systematic) review of literature. Weighing of the evidence and recommendations adjusted or added if necessary.

In the Dutch guideline program for physical therapy patients and other disciplines are included in the external review group. This approach allows the development group to focus primarily on the physiotherapy process, while specific input from patients and other disciplines is ensured in the external review. As a result, the updated procedure complied with 22 AGREE criteria (Table 3).

## Conclusion

As a result of international developments and consensus, the described processes for developing clinical practice guidelines have much in common. The AGREE criteria are common basis for the development processes in the different guideline programs. We learned that prioritizing topics, defining the scope of the guideline and the formulation of recommendations can be more clearly described. In particular, the process of translation of evidence into practice recommendations can be improved. The previous KNGF program for guideline development in physical therapy lacked compliance with the AGREE criteria. Detailed comparison of the different guideline programs was used for updating the KNGF guideline program, which reflects international consensus and describes explicit choices in those issues lacking consensus. As a result the updated KNGF program complied with 22 AGREE criteria. International discussion is continuing and will be used for further improvement of the program.

## Competing interests

The author(s) declare that they have no competing interests.

## Authors' contributions

PW and EH collected data and analyzed the guideline programs. PW, EH and JC updated the Dutch program for guideline development. JD, RB and JB critically revised the manuscript and contributed to the international perspective of the study. All authors read and approved the final manuscript.

## Pre-publication history

The pre-publication history for this paper can be accessed here:


